# Prognostic implications of a CD8^+^ T_EMRA_ to CD4^+^T_reg_ imbalance in mandibular fracture healing: a prospective analysis of immune profiles

**DOI:** 10.3389/fimmu.2024.1476009

**Published:** 2024-10-23

**Authors:** Jan Oliver Voss, Fabio Pivetta, Aboelyazid Elkilany, Katharina Schmidt-Bleek, Georg N. Duda, Kento Odaka, Ioanna Maria Dimitriou, Melanie Jasmin Ort, Mathias Streitz, Max Heiland, Steffen Koerdt, Simon Reinke, Sven Geissler

**Affiliations:** ^1^ Charité – Universitätsmedizin Berlin, corporate member of Freie Universität Berlin, Humboldt-Universität zu Berlin, and Berlin Institute of Health, Department of Oral and Maxillofacial Surgery, Berlin, Germany; ^2^ Berlin Institute of Health at Charité – Universitätsmedizin Berlin, BIH Academy, Clinician Scientist Program, Berlin, Germany; ^3^ Charité – Universitätsmedizin Berlin, corporate member of Freie Universität Berlin, Humboldt-Universität zu Berlin and Berlin Institute of Health, Department of Radiology, Berlin, Germany; ^4^ Berlin Institute of Health at Charité – Universitätsmedizin Berlin, Julius Wolff Institute (JWI), Berlin, Germany; ^5^ Berlin Institute of Health at Charité Universitätsmedizin, BIH Center for Regenerative Therapies (BCRT), Berlin, Germany; ^6^ Department of Oral and Maxillofacial Radiology, Tokyo Dental College, Chiyoda-Ku, Tokyo, Japan; ^7^ Freie Universität Berlin, Institute for Chemistry and Biochemistry, Berlin, Germany; ^8^ Friedrich-Loeffler-Institute, Federal Research Institute for Animal Health, Greifswald – Insel Riems, Germany

**Keywords:** ossification, mandibular fractures, bone healing, non-union, pseudarthrosis, TEMRA

## Abstract

**Introduction:**

Open reduction and fixation are the standard of care for treating mandibular fractures and usually lead to successful healing. However, complications such as delayed healing, non-union, and infection can compromise patient outcomes and increase healthcare costs. The initial inflammatory response, particularly the response involving specific CD8^+^ T cell subpopulations, is thought to play a critical role in healing long bone fractures. In this study, we investigated the role of these immune cell profiles in patients with impaired healing of mandibular fractures.

**Materials and methods:**

In this prospective study, we included patients with mandibular fractures surgically treated at Charité – Universitätsmedizin Berlin, Germany, between September 2020 and December 2022. We used follow-up imaging and clinical assessment to evaluate bone healing. In addition, we analyzed immune cell profiles using flow cytometry and quantified cytokine levels using electrochemiluminescence-based multiplex immunoassays in preoperative blood samples.

**Results:**

Out of the 55 patients enrolled, 38 met the inclusion criteria (30 men and 8 women; mean age 32.18 years). Radiographic evaluation revealed 31 cases of normal healing and 7 cases of incomplete consolidation, including 1 case of non-union. Patients with impaired healing exhibited increased levels of terminally differentiated effector memory CD8^+^ T cells (T_EMRA_) and a higher T_EMRA_ to regulatory T cell (T_reg_) ratio, compared with those with normal healing.

**Conclusions:**

Our analysis of mandibular fracture cases confirms our initial hypothesis derived from long bone fracture healing: monitoring the T_EMRA_ to T_reg_ ratio in preoperative blood can be an early indicator of patients at risk of impaired bone healing. Radiologic follow-up enabled us to detect healing complications that might not be detected by clinical assessment only. This study highlights the potential of individual immune profiles to predict successful healing and may form the basis for future strategies to manage healing complications.

## Introduction

Mandibular fractures are among the most common fractures of the facial skeleton (1). The treatment strategies are primarily conservative, such as observation and closed treatment with maxillary-mandibular fixation or functional therapy in non- or minimal displaced fractures of the condylar process and head. However, in cases of multi-fragmentary fractures, severe dislocation of fracture fragments, and fractures in the tooth-bearing mandibular body, surgical treatment is required (2).

The healing of bone structures, including mandibular fractures, typically results in complete restoration of structure and function without scarring (3), indicating the marked endogenous healing capability of bones. In some patients, however, major complications may arise, such as wound-healing problems, infection, osteomyelitis, delayed healing, and non-union (4, 5). The incidence of such complications can reach up to 21%, albeit with variations based on the patient population and the inclusion criteria (6–9). Previous studies have indicated that there is delayed healing in approximately 9.5% of cases, as well as occurrences of non-union, pseudarthrosis, and osteomyelitis in up to 5% of cases (10–13). Inadequate fracture treatment can lead to postoperative osteomyelitis and non-union, necessitating revision surgery and refixation (14). Non-union fractures alone require supplementary procedures, resulting in extended hospitalization and prolonged rehabilitation periods. The associated healthcare costs are more than 30% higher than those for uncomplicated cases, and these fractures can significantly impact health-related quality of life (15, 16).

A clinical consensus regarding the definition and assessment of delayed or incomplete fracture healing, especially in the mandible, remains elusive. The evaluation of mandibular healing primarily relies on clinical observations, supplemented by cross-sectional or secondary conventional imaging when impairment is suspected. Successful healing is typically inferred based on healing progression in sequential radiographs or increased mechanical stability. The subjective nature of clinical signs, such as pain and loss of function, coupled with the surgeon’s experience, can influence the quality of the assessment (3). While there are some classification systems for non-union fractures in long bones, there is no standardized scoring system for non-union in mandibular fractures (17, 18). Defining the appropriate healing time and diagnosing non-healing are also complicated by variables such as the fracture type and comorbidities.

There are several factors that can impair mandibular healing after fracture treatment, including tobacco and alcohol use, age, preexisting conditions, the treatment modality, and the fracture location and pattern (4, 6, 7, 9, 13, 19–22). Furthermore, the patient’s immune system seems to play a critical role in bone repair and the healing outcome (23, 24). Studies on long bones in mice have shed light on the intricate interaction between T and B cells and osteoblasts and osteoclasts at the fracture site (25). El Khassawna et al. (26) found dysregulation in collagen deposition in mice lacking mature T and B cells, suggesting a role for these cells in the mineralization process of bone formation after a fracture. Furthermore, different T cell subsets can have distinct impacts on the healing process based on individual immune experiences (27). Additionally, the cytokines released from immune cells significantly influence healing (28).

An unresolved issue is whether findings regarding the healing processes in long bones can be extrapolated to facial bones such as the mandible. The unique anatomy and biomechanics of facial bones may manifest different healing patterns and responses to treatment compared with those of the long bones (29). In contrast to closed fractures of the long bones, fractures in the tooth-bearing part of the mandible are consistently exposed to pathogens in the oral cavity through the periodontal space. Of note, the mandible, which is constantly subjected to masticatory forces, may necessitate distinct assessment criteria and longer healing durations than the long bones, which primarily endure weight-bearing loads. Even from an embryological standpoint, there are evident disparities between long bones, which develop primarily via endochondral ossification, and the mandible and most cranial bones, which undergo primarily intramembranous ossification. This fundamental difference complicates the direct application of the findings from long-bone studies to the mandible (30, 31). A comprehensive understanding of these variances is essential for precise diagnosis and treatment planning, and could potentially unveil opportunities for the early identification of patients at increased risk of healing complications.

Currently, there is no early prediction method for high-risk patients in oral and maxillofacial surgery, which underscores an unmet medical need. Such an approach could facilitate timely interventions or the application of adjunctive procedures to support anticipated compromised mandibular healing. Therefore, we aimed to determine whether distinct T cell profiles in patients with mandibular fractures correlate with their healing outcomes.

## Patients and methods

### Ethics statement

This study was conducted in compliance with the International Conference on Harmonization Guidelines for Good Clinical Practice and the Declaration of Helsinki. Ethical permission for data collection and publication was granted by the Institutional Review Board of Charité – Universitätsmedizin Berlin (EA2/107/20). Prior to inclusion in the study, each participating patient provided written informed consent.

### Subjects and study design

This monocentric, prospective study investigated bone healing after mandibular fracture in patients treated from September 2020 to December 2022 at the Department of Maxillofacial Surgery of Charité – Universitätsmedizin Berlin, Germany. The inclusion criteria were age ≥ 18 years, capable of providing informed consent, and a mandibular fracture affecting the tooth-bearing part of the mandible (body, median, paramedian, or angle) treated by osteosynthesis plating (open reduction and internal fixation [ORIF]). Pregnant patients were excluded from the study. Only patients who underwent preoperative peripheral blood sampling as well as preoperative and follow-up imaging could be included in the final analysis.

Peripheral pre- and postoperative blood samples, as well as fracture hematoma from the mandibular fracture site, were collected for further analysis. Postoperative blood samples were collected between days 1 and 3 after surgery. Patients who requested the removal of postoperative material received additional imaging after 6–9 months (panoramic imaging or cone-beam computed tomography [CBCT]) and were further analyzed. Due to the heterogeneous nature of postoperative imaging, different imaging/X-ray modalities, including panoramic imaging, CBCT, and CT, were used.

### Assessment of the mandibular fracture gap in follow-up imaging after ORIF

**Figure 1:**
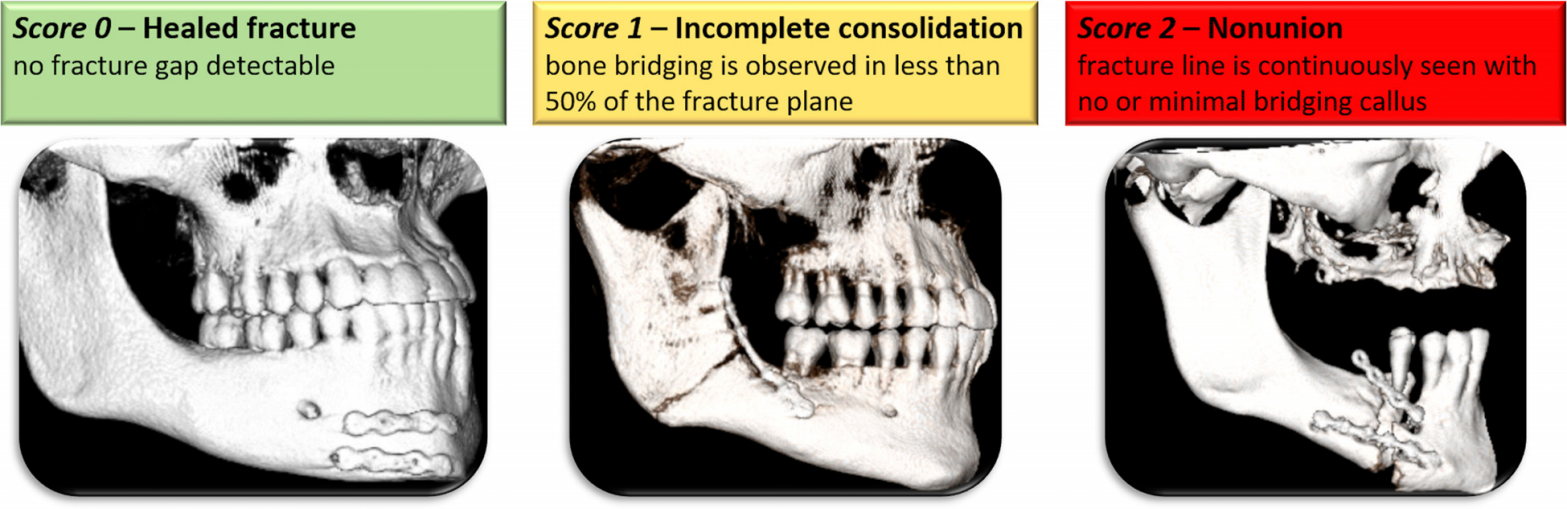
An illustration of the scoring system to evaluate bone healing based on follow-up imaging, with a score of 0 on the left side and a score of 2 on the right side.

Each patient included in the final analysis underwent radiological follow-up imaging to assess changes in the fracture gap over time. This follow-up imaging was performed at least 6 months after fracture repair or earlier if there was a clinical complaint. Radiological imaging was examined by three independent, blinded radiologists. These radiologists rated the fracture gap in the follow-up images by using the following scoring system (**Figure 1**):

1. *Score 0:* no fracture gap detectable, complete fracture consolidation.
2. *Score 1:* a fracture gap is partially detectable, indicating incomplete consolidation (bone bridging observed in < 50% of the fracture plane).
3. *Score 2:* a fracture gap is completely detectable, indicating non-union (a fracture line is seen throughout, and no or a minimal bridging callus is present).

### Sample collection, processing, and flow cytometry analysis

Pre- and postoperative peripheral blood samples were collected in vacutainers containing ethylenediaminetetraacetic acid (EDTA) (for blood counts) or heparin lithium (to isolate plasma) and stored at room temperature for further analysis. From the EDTA blood samples, 150 µL of whole blood was taken for subsequent fluorescence-activated cell sorting (FACS) staining. The remaining whole blood was centrifuged for 10 minutes at 2000 g, and the resulting supernatant was stored at −80°C. Fracture hematomas were collected intraoperatively and stored in a vacutainer containing EDTA and 0.9% sodium chloride. A gentleMACS™ dissociator (Miltenyi Biotec, Bergisch Gladbach, Germany) was used to generate a single-cell suspension; subsequently, it was filtered through a Falcon 100-µm nylon cell strainer (Thermo Fisher Scientific, Waltham, MA, USA). The cell suspension was stained by following the DURAClone IM T_reg_ and IM T cell protocols provided by the manufacturer (Beckman Coulter, Brea, CA, USA).

All samples were further processed according to the manufacturer’s recommendations/instructions. For immunophenotyping, cell surface markers in the EDTA blood samples were labeled using fluorescently conjugated antibodies to T cell–related cell surface protein markers. The DURAClone IM T cell subset kit includes CD45RA (CD4-fluorescein isothiocyanate), CD197/CCR7 (CD4-phycoerythrin), CD28 (CD28- phycoerythrin-Texas Red-X), CD279/PD1 (phycoerythrin-Cyanine 5.5), CD27 (phycoerythrin-Cyanine 7), CD4 (allophycocyanin), CD8 (Alexa Fluor 700), CD3 (allophycocyanin-Alexa Fluor 750), CD57 (Pacific Blue), and CD45 (Krome Orange). The DURAClone IM T_reg_ subset kit includes CD45RA (fluorescein isothiocyanate), CD35 (phycoerythrin), CD39 (phycoerythrin-cyanine 5.5), CD4 (phycoerythrin-Cyanine 7), FoxP3 (Alexa Fluor 647), CD3 (allophycocyanin-Alexa Fluor 750), Helios (Pacific Blue), and CD45 (Krome Orange).

A Navios EX Flow instrument (Beckman Coulter) was used for flow cytometry acquisition (10 colors, 3 lasers). The flow cytometer was calibrated using calibrator beads according to the manufacturer’s instructions. The gating strategy for memory T cell subsets and T cell activation is presented in **Supplementary Figure S1**. The gating strategy for T cells (T_regs_) is shown in **Supplementary Figure S2**. The Kaluza Analysis 2.1 Software (Beckman Coulter) was used for data analysis.

### Biomarker quantification

Biomarkers were quantified in preoperative serum samples using MESO QuickPlex SQ 120 mm technology (Meso Scale Diagnostics, Rockville, MD, USA), an electrochemiluminescence-based multiplex immunoassay. Here, a customized panel (U-PLEX, Custom Metabolic Group 1) was used to quantify 10 different factors, namely interleukin 1beta (IL-1β), B cell activating factor (BAFF), beta-nerve growth factor (βNGF), fibroblast growth factor 23 (FGF-23), IL-6, IL-8, IL-10, leptin, monocyte chemoattractant protein 1 (MCP-1), and tumor necrosis factor alpha (TNF-α). All samples were diluted 1:2 and measured in duplicate on each assay plate.

In brief, the sandwich principle was followed: Each well in a U-plate includes up to 10 spots, each of which is specific for an analyte and captures the respective U-plex linker. The U-plex linkers are coupled to biotinylated antibodies that then bind to the sample. Finally, the SULFO-TAG™-conjugated detection antibodies are added and emit light once the appropriate excitation wavelength is applied to the plate electrodes. The concentration of each analyte in the sample can be calculated based on the intensity of emitted light, as these metrics (parameters) are proportional. The final concentrations are presented in pg/mL.

Calibrators were used to construct a standard curve for each analyte. Each vial of calibrator was reconstituted according to the manufacturer’s instructions, after reaching room temperature, resulting in a 10× concentrated stock solution. Then, this stock solution was diluted 10-fold to establish the highest concentration point on the standard curve, referred to as Calibrator Standard 1. Subsequently, six additional 4-fold serial dilutions were prepared, with the final dilution serving as Calibrator Standard 8 (zero Calibrator/Blank). The lower limit of detection (LLOD) was defined as 2.5 times the standard deviation above the background signal (blank control). This value was computed by the analysis software for each analyte (**Table 1**).

**Table 1:** The lower limit of detection (LLOD) and the upper limit of detection (ULOD) for each analyte [pg/ml].

Biomarker	LLOD	ULOD
BAFF	0.0684	476
FGF-23	0.343	2550
IL-6	0.239	980
IL-8	0.0952	1160
IL-10	0.152	2030
IL-1β	0.174	2415
Leptin	5.61*	48800
βNGF	0.0603	445
TNFα	0.156	1505
MCP-1	0.430	3815

*The leptin level in patient 1 was below the detection limit.

### Statistical analysis

GraphPad Prism Version 9.20 (GraphPad Software, Boston, MA, USA) was used for statistical analysis. The Kolmogorov–Smirnov test with a Dallal–Wilkinson–Lillie-corrected *P* value was used to assess whether the data followed a Gaussian distribution. Two groups were compared with a *t*-test (parametric data) or the Mann–Whitney U-test (non-parametric data). Pearson correlation analysis was used for parametric data, and Spearman correlation analysis was used for non-parametric data. A paired *t*-test was used to analyze pre- and postoperative blood samples from the same patient. The inter-rater agreement was assessed based on Fleiss’ kappa. The results are rounded to two decimal places, and *P* < 0.05 was defined as statistically significant. Fisher’s exact test was used to test for contingency.

Receiver operating characteristic (ROC) curves were generated to assess the diagnostic performance of the selected immune cell markers in predicting the bone healing outcome. The markers were categorized by healing status (normal vs. impaired), and sensitivity and the false positive rate were calculated across the threshold values. The area under the curve (AUC) measured each marker’s discriminatory ability, with values near 1 indicating high accuracy. Confidence intervals were calculated to determine the significance of the AUC, and optimal thresholds were identified to balance sensitivity and specificity.

## Results

We enrolled a cohort of 55 patients, of whom 38 (30 men and 8 women) met the prespecified inclusion criteria, which included preoperative blood sampling and subsequent follow-up imaging (**Supplementary Figure S3**). These patients had a total of 68 fracture sites, with 43 being relevant to our analysis. The mean age of the patient cohort was 32 ± 12 years, ranging from 18 to 66 years. The most common causes of mandibular fractures were assaults (n = 20) and bicycle or e-bike incidents (n = 11). **Table 2** provides a comprehensive overview of the demographics and relevant characteristics of the patients.

**Table 2:** Overview of the patients’ characteristics.

		Normal healing (n = 31)	Impaired healing (n = 7)	Overall	*P* value
Age in years (mean ± standard deviation)		31.26 ± 12.45	36.29 ± 10.21	32.18 ± 12.10	0.136
Gender (n)	Male	23	7	30	0.310
	Female	8	0	8	
Substance abuse (n)	Nicotine	10	3	13	0.670
	Alcohol	11	2	13	1.000
Etiology (n)	Assault	15	5	20	
	Fall	3	1	4	
	Bike/e-bike accident	10	0	10	
	Activities of daily living	3	1	4	
Follow-up in weeks (mean ± standard deviation)		32.73 ± 10.11	24.73 ± 5.78	31.26 ± 9.9	0.013
Clinical complaints (total events)		0	2	2	
Radiological score	0	31	0	31	
	1	0	6	6	
	2	0	1	1	

Approximately 95% (n = 36) of follow-up clinical consultations were uneventful. The mean interval between surgery and follow-up imaging was 31 ± 10 weeks. Radiological assessment by three independent radiologists revealed complete bone healing in 31 cases and incomplete fracture consolidation in 7 cases, with an inter-rater agreement (Fleiss’ kappa) of 0.31. Two patients with incomplete consolidation also experienced other complications: One had pain-associated mobility at the fracture site, and the other had exposed osteosynthesis material. Both cases required additional surgery: one for early plate removal due to infection and the other for re-osteosynthesis due to non-union.

Based on these assessments, we categorized the patients into two groups, namely normal healing (n = 31; 81.5%) and impaired healing (n = 7; 18.5%). All patients with impaired bone healing were men (odds ratio 5.43, 95% confidence interval [CI] 0.29–105.6, *P* = 0.31). There was no significant association between smoking (*P* = 0.67) or alcohol abuse (*P* = 1.00) and impaired bone healing. The mean age of the patients with impaired bone healing was 36.3 ± 10.2 years (median = 35 years), compared with 31.3 ± 12.5 years (median = 26 years) in patients with normal bone healing, but this difference was not significant.

**Figure 2:**
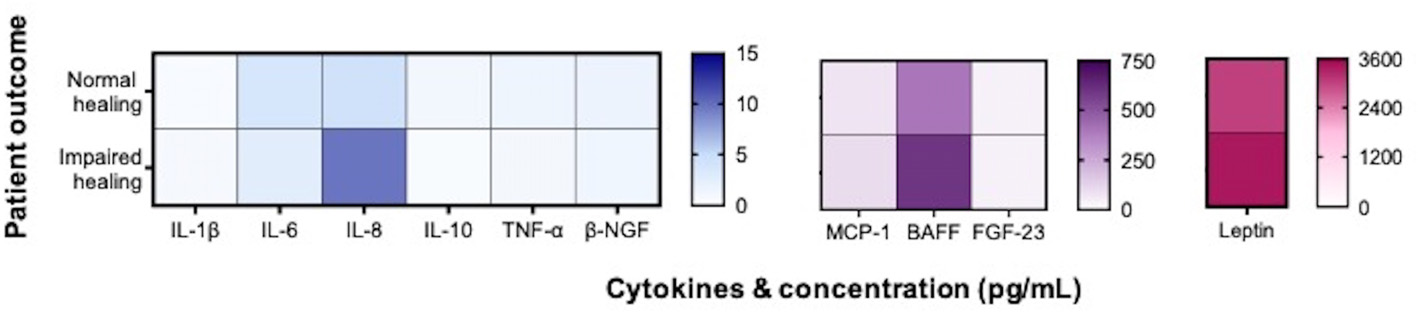
Aheatmap of 10 metabolic and pro-inflammatory markers in patient serum. The data were divided into two groups based on the healing outcome: normal healing (n = 20) and impaired healing (n = 6). The mean cytokine levels (in pg/mL) are presented for each group.

Next, we investigated whether the fracture induces a detectable systemic inflammatory response that correlates with the healing outcome. Specifically, we analyzed surrogate marker profiles in patient serum samples by using a multiplex immunoassay. We found no significant differences in the preoperative levels of IL-1β, BAFF, β-NGF, FGF-23, IL-6, IL-8, IL-10, leptin, MCP-1, and TNF-α between the two groups (**Figure 2**, **Supplementary Figure S4**).

**Figure 3:**
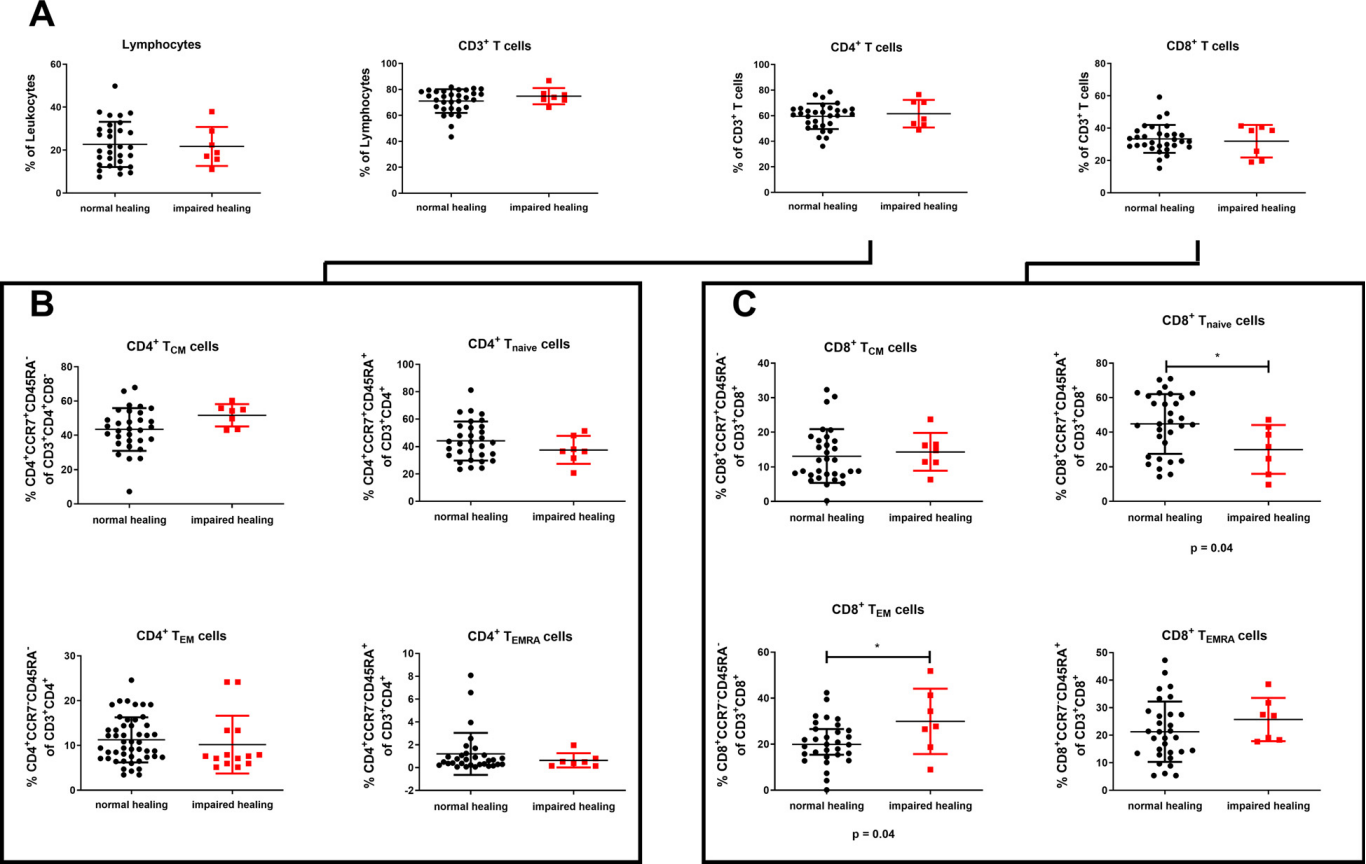
Delayed bone healing is linked to a more experienced systemic adaptive immune profile. The distributions of different immune cell populations with regard to bone healing in patients with mandibular fractures are shown. **(A)** Lymphocytes, CD3^+^ T cells, CD4^+^ T cells, and CD8^+^ T cells. **(B)** CD4^+^ T cell subsets: central memory (CD4^+^ T_CM_, CCR7^+^CD45RA^–^), naive (CD4^+^ T_naïve_, CCR7^+^CD45RA^+^), effector memory (CD4^+^ T_EM_, CCR7^–^CD45RA^–^), and terminal differentiated effector memory (CD4^+^ T_EMRA_, CCR7^–^CD45RA^+^). **(C)** CD8^+^ T cell subsets: central memory (CD8^+^ T_CM_, CCR7^+^CD45RA^–^), naive (CD8^+^ T_naïve_, CCR7^+^CD45RA^+^), effector memory (CD8^+^ T_EM_, CCR7^–^CD45RA^–^), and terminal differentiated effector memory (CD8^+^ T_EMRA_; CCR7^–^CD45RA^+^). The data are presented as the mean ± standard deviation of the indicated group (n = 38). For A–C, an unpaired two-sided *t*-test was used for statistical analysis. *P < 0.05.

To investigate potential correlations between the healing outcome and distinct adaptive immune profiles, we examined the systemic T cell composition in the preoperative blood samples. Our analysis revealed no significant differences in lymphocyte distribution, including CD3^+^, CD4^+^, and CD8^+^ T cells, between patients with impaired and normal bone healing (**Figure 3A**). Subsequent analysis of CD4^+^ T cell subpopulations showed no detectable difference between the two groups (**Figure 3B**). However, there were notable variations within the CD8^+^ T cell population. Patients exhibiting normal bone healing displayed higher mean levels of naïve CD8^+^ T cells (T_naïve_, CCR7^+^CD45RA^+^) than those with impaired healing (*P* = 0.04). Conversely, patients with impaired bone healing exhibited elevated levels of effector memory T cells (T_EM_, CCR7^-^CD45RA^-^) (*P* = 0.04, **Figure 3C**). Furthermore, while there was trend for a higher mean level of terminally differentiated effector memory CD8^+^ T cells (T_EMRA_; CCR7^–^CD45RA^+^) in patients with impaired bone healing, the difference was not significant (*P* = 0.32, **Figure 3C**). Nonetheless, these findings suggest that patients with delayed healing have more experienced adaptive immunity than those with normal fracture healing.

**Figure 4:**
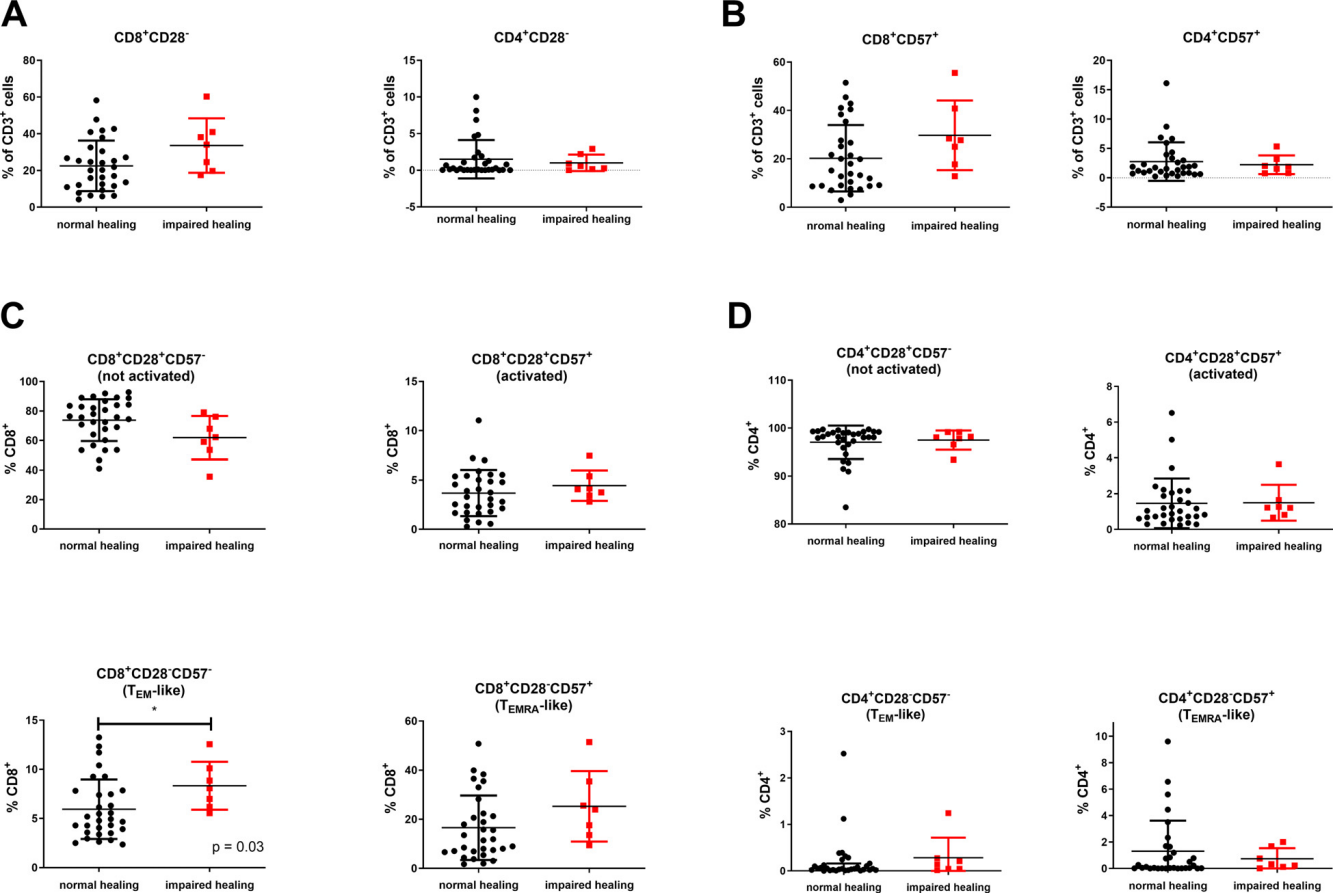
Elevated systemic levels of (CD28^–^CD57^–^) CD8^+^ T_EM_-like and (CD28^–^CD57^+^) CD8^+^ T_EMRA_-like cells in patients with compromised bone healing. **(A)** CD8^+^CD28^–^ and CD4^+^CD28^–^ T cells. **(B)** CD8^+^CD57^+^ and CD4^+^CD57^+^ T cells. **(C)** Non-activated (CD28^+^CD57^–^) CD8^+^ T cells, activated (CD28^+^CD57^+^) CD8^+^ T cells, effector memory-like (T_EM_-like) (CD28^–^CD57^–^) CD8^+^ T cells, and terminal differentiated effector memory-like (T_EMRA_-like) (CD28^–^CD57^+^) CD8^+^ T cells. **(D)** Non-activated (CD28^+^CD57^–^) CD4^+^ T cells, activated (CD28^+^CD57^+^) CD4^+^ T cells, (CD28^–^CD57^–^) CD4^+^ T_EM_-like cells, and (CD28^–^CD57^+^) CD4^+^ T_EMRA_-like cells. The data are presented as the mean ± standard deviation of the indicated group (n = 38). For **(A, B, D)**, an unpaired two-sided *t*-test was used for statistical analysis. For **(C)**, the Mann–Whitney U test was employed for statistical analysis. *P < 0.05.

An alternative approach to distinguish memory and terminally differentiated CD8^+^ T cell subsets involves evaluating the expression of CD28 and CD57 (32). Accordingly, four distinct CD8^+^ T-cell subsets can be identified: non-activated CD28^+^CD57^−^, activated CD28^+^CD57^+^, activated or T_EM_-like CD28^−^CD57^−^, and T_EMRA_-like CD28^−^CD57^+^ CD8^+^ T cells. The levels of CD28^–^CD8^+^ T cells (*P* = 0.07) and CD57^+^ CD8^+^ T cells (*P* = 0.08) tended to be lower in patients with normal bone healing; however, these differences did not reach statistical significance (**Figures 4A, B**). While patients with normal healing exhibited comparable levels of circulating activated (CD28^+^CD57^+^) CD8^+^ T cells to patients with impaired healing (*P* = 0.42), their systemic levels of non-activated (CD28^+^CD57^–^) CD8^+^ T cells were slightly higher (although the difference was not significant, *P* = 0.07, **Figure 4C**). Conversely, patients with impaired healing demonstrated significantly elevated levels of (CD28^–^CD57^–^) CD8^+^ T_EM_-like cells (*P* = 0.03, **Figure 4C**) and (CD28^–^CD57^+^) CD8^+^ T_EMRA_-like cells (*P* = 0.08, **Figure 4C**). We also explored the expression of CD28 and CD57 within the CD8^+^CCR7^–^ T cell subset to better understand the relationship between immune experience and the fracture healing outcome. We found that the CD8^+^CCR7^–^ effector memory compartment was significantly elevated in patients with impaired fracture healing compared with those with normal healing (*P* = 0.027, **Supplementary Figure S5**). Consistent with our previous observations, patients with impaired healing also exhibited elevated levels of CD8^+^CCR7^–^CD28^–^CD57^–^ T_EM_-like cells (*P* = 0.032) and CD8^+^CCR7^–^CD28^–^CD57^+^ T_EMRA_-like cells (*P* = 0.064) (**Supplementary Figure S5**). However, the percentage of CD8^+^CCR7^–^,CD28^–^CD57^–^, and CD8^+^CD28^–^CD57^–^ T_EM_-like were comparable within the CD8^+^ T cell subset (normal healing: mean [CCR7^–^CD28^–^CD57^–^] = 5.5% and mean [CD28^–^CD57^–^] = 5.9%, *P* = 0.557; impaired healing: mean [CCR7^–^CD2^–^CD57^+^] = 8.1% and mean [CD28^–^CD57^+^] = 8.3%, *P* = 0.877). Similarly, in each group there was not a significant difference between the levels of CD8^+^CCR7^–^CD28^–^CD57^–^ and CD8^+^CD28^–^CD57^-^ T_EMRA_-like cells (normal healing: *P* = 0.727; impaired healing: *P* > 0.999). In contrast, there were significant differences in the levels of CD8^+^CCR7^–^CD28^+^CD57^–^ and CD8^+^CD28^+^CD57^–^ (non-activated) T cells, independent of the healing outcome (normal healing: mean [CCR7^–^CD28^+^CD57^–^] = 18.8%, mean [CD28^+^CD57^–^] = 73.8%, *P* < 0.001; impaired healing: mean [CCR7^–^CD28^–^CD57^+^] = 19.2%, mean [CD28^–^CD57^+^] = 61.9%, *P* = 0.001, **Supplementary Figure S5**). Based on the results, these subpopulations are more closely associated with naive CD8^+^ T cells, again indicating a potential link between a more experienced immune system and impaired bone healing.

**Figure 5:**
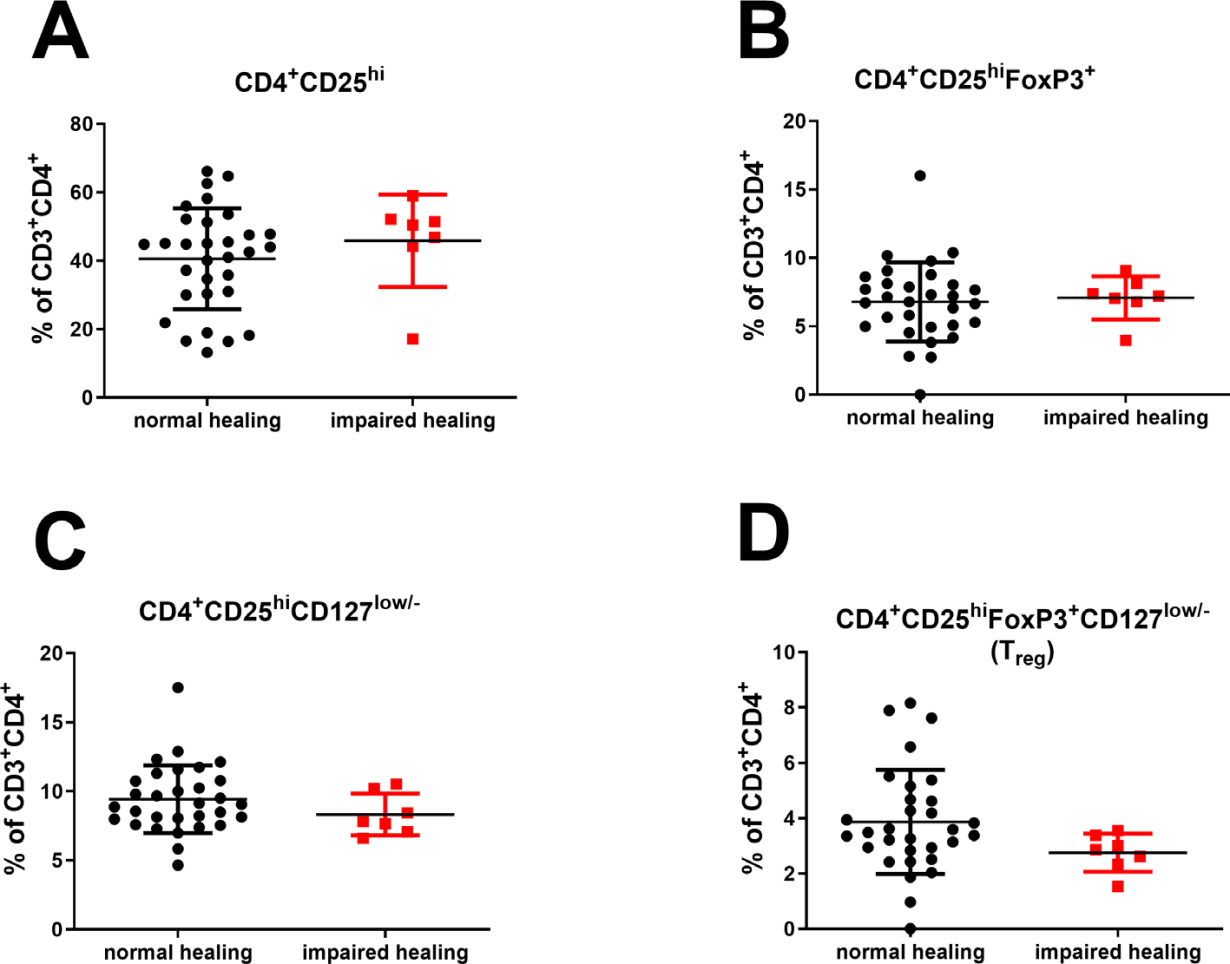
No significant differences in systemic CD4^+^ T_reg_ levels between normal and impaired healing. **(A)** CD4^+^CD25^hi^ T cells. **(B)** CD4^+^CD25^hi^FoxP3^+^ T cells. **(C)** CD4^+^CD25^hi^CD127^low/–^ T cells. **(D)** CD4^+^CD25^hi^FoxP3^+^CD127^low/–^ T cells. The data are presented as the mean ± standard deviation of the indicated group (n = 38).

Characterization of CD4^+^ T cells using CD28 and CD57 revealed that the majority of CD4^+^ T cells were CD28^+^ or CD57^–^ (**Figure 4D**). Accordingly, we detected only low levels of CD28^+^CD57^–^, CD28^–^CD57^–^, and CD28^–^CD57^+^ CD4^+^ T cells, with no significant differences between the two groups. Given previous findings on the regulatory role of CD4^+^ T_regs_ in inflammation and bone regeneration (33), we also evaluated the levels of this subset in the blood samples. CD4^+^ T_regs_ are characterized by high expression of CD25 and FoxP3, by high expression of CD25 and low levels of CD127, or a combination of both. We found no significant differences in CD4^+^CD25^hi^ or CD4^+^CD25^hi^FoxP3^+^ T cell levels between normal and impaired healing. Although CD4^+^CD25^hi^CD127^low/–^ and CD4^+^CD25^hi^FoxP3^+^CD127^low/–^ T cell levels were reduced in the blood samples of patients with impaired healing, the difference was not significant (*P* = 0.055; **Figure 5**).

**Figure 6:**
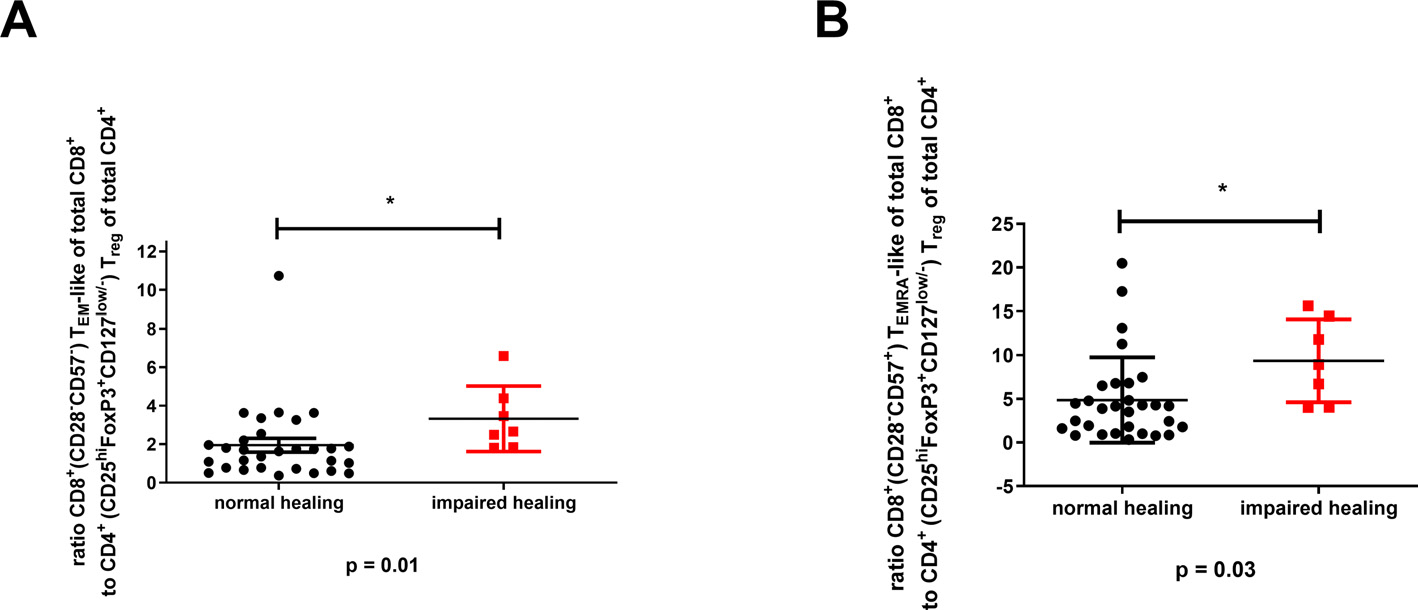
Significant association between the (CD28^–^CD57^–^) CD8^+^ T_EM_-like or (CD28^–^CD57^+^) CD8^+^ T_EMRA_-like cell to (CD25^hi^FoxP3^+^CD127^low/–^) CD4^+^ T_reg_ ratios and impaired bone healing. **(A)** The CD8^+^ T_EM_-like cell to CD4^+^ T_reg_ ratio. **(B)** The CD8^+^ T_EMRA_ cell to CD4^+^ T_reg_ ratio. The data are presented as the mean ± standard deviation for the indicated group (n = 38). For **(A, B)**, an unpaired two-sided *t*-test was used for statistical analysis. *P < 0.05.

However, the elevated levels of (CD28^–^CD57^–^) CD8^+^ T_EM_-like and (CD28^–^CD57^+^) CD8^+^ T_EMRA_-like cells, along with reduced (CD25^hi^FoxP3^hi^CD127^low/–^) CD4^+^ T_reg_ levels in patients with impaired bone healing, resulted in significantly higher CD8^+^ T_EM_-like to CD4^+^ T_reg_ (*P* = 0.01) and CD8^+^T_reg_T_EMRA_-like to CD4^+^ T_reg_ ratios (*P* = 0.03) in this patient group (**Figure 6**). In addition, we observed significant increases in the ratios of (CCR7^–^CD45RA^–^) CD8^+^ T_EM_ cells to CD4^+^ T_regs_ and (CCR7^–^CD45RA^+^) CD8^+^ T_EMRA_ cells to CD4^+^ T_regs_ in patients with impaired healing (**Supplementary Figure S6**). Taken together, these findings suggest potential immune dysregulation that may impact bone repair processes. We attempted to confirm these findings by analyzing T cell profiles in the fracture hematomas. However, due to the typically small volume of hematomas, this assessment was only possible in six cases. This limited sample size hindered the quantification of T cell subpopulations, including CD4^+^ T_regs_ and CD8^+^ T_EMRA_-like cells, preventing statistical evaluation and comparisons regarding bone healing.

Because immune experience and the fracture healing outcome might be linked to aging, we specifically examined age as a potential confounder. Our analysis showed no significant association between age and the fracture healing outcome (*P* = 0.1363, **Table 1**). Except for CD8^+^ (CCR7^–^CD45RA^–^) T_EM_ cells, which were significantly associated with age (Spearman r = 0.432, p = 0.006), there were no significant relationships between age and the levels of CD8^+^ (CD28^–^CD57^–^) T_EM_-like cells (Spearman r = 0.122, *P* = 0.465), CD8^+^ (CCR7^–^CD45RA^+^) T_EMRA_ cells (Spearman r = 0.051, *P* = 0.759), or CD8^+^ (CD28^–^CD57^+^) T_EMRA_-like cells (Spearman r = 0.261, *P* = 0.113; **Supplementary Figure S7**). Furthermore, there were no significant associations between age and the ratios of CD8^+^ T_EM_ or T_EMRA_ cells to CD4^+^ T_regs_ (all *P* > 0.05; **Supplementary Figure S7**). These findings suggest that the impaired healing associated with elevated CD8^+^ T_EM_ and T_EMRA_ cell levels, as well as their ratios to CD4^+^ T_regs_, is not directly related to the patient’s age.

**Figure 7:**
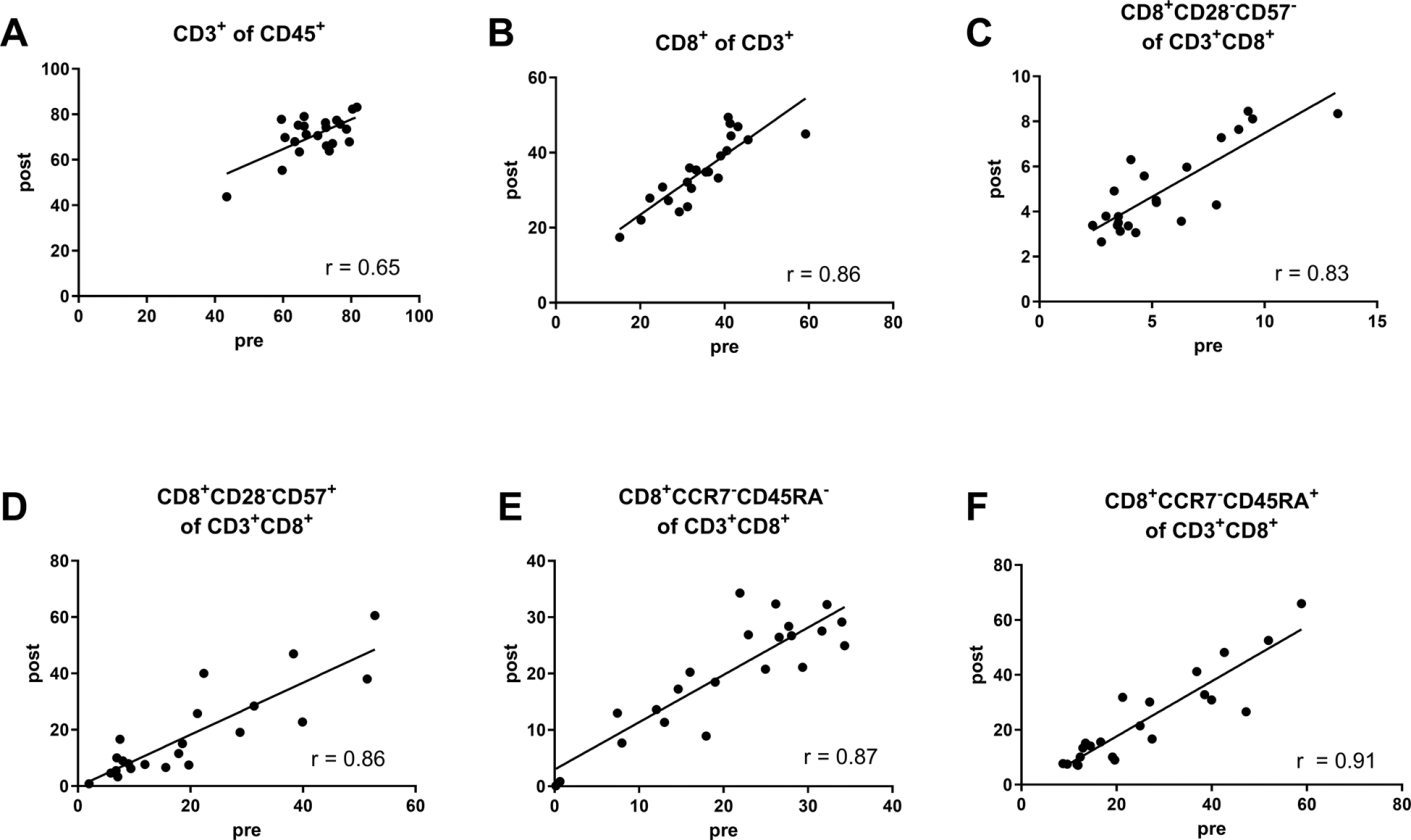
Correlation analysis, reveals there are no significant differences in marker stability across various CD8^+^ T cell subsets. **(A)** CD3^+^ T cells as a percent of CD45^+^ cells (Pearson r = 0.65, 95% CI 0.32–0.84, *P* = 0.001). **(B)** CD8+ T cells as a percent of CD3+ T cells (Pearson r = 0.86, 95% CI 0.68–0.94, *P* < 0.0001). **(C)** CD28^–^CD57^–^ CD8^+^ T_EM_-like cells as a percent of CD3^+^CD8^+^ T cells (Pearson r = 0.83, 95% CI 0.63–0.93, *P* < 0.0001). **(D)** CD28^–^CD57^+^ CD8^+^ T_EMRA_-like cells as a percent of CD3^+^CD8^+^ T cells (Pearson r = 0.86, 95% CI 0.69–0.94, *P* < 0.0001). **(E)** CCR7^–^CD45RA^–^ CD8^+^ T_EM_ cells as a percent of CD3^+^CD8^+^ T cells (Pearson r = 0.87, 95% CI 0.70–0.95, *P* < 0.0001). **(F)** CCR7^–^CD45RA^+^ CD8^+^ T_EMRA_ cells as a percent of CD3^+^CD8^+^ T cells (Pearson r = 0.91, 95% CI 0.79–0.96, *P* < 0.0001). The data are based on the pre- and postoperative blood samples from the same patients (n = 22).

**Figure 8:**
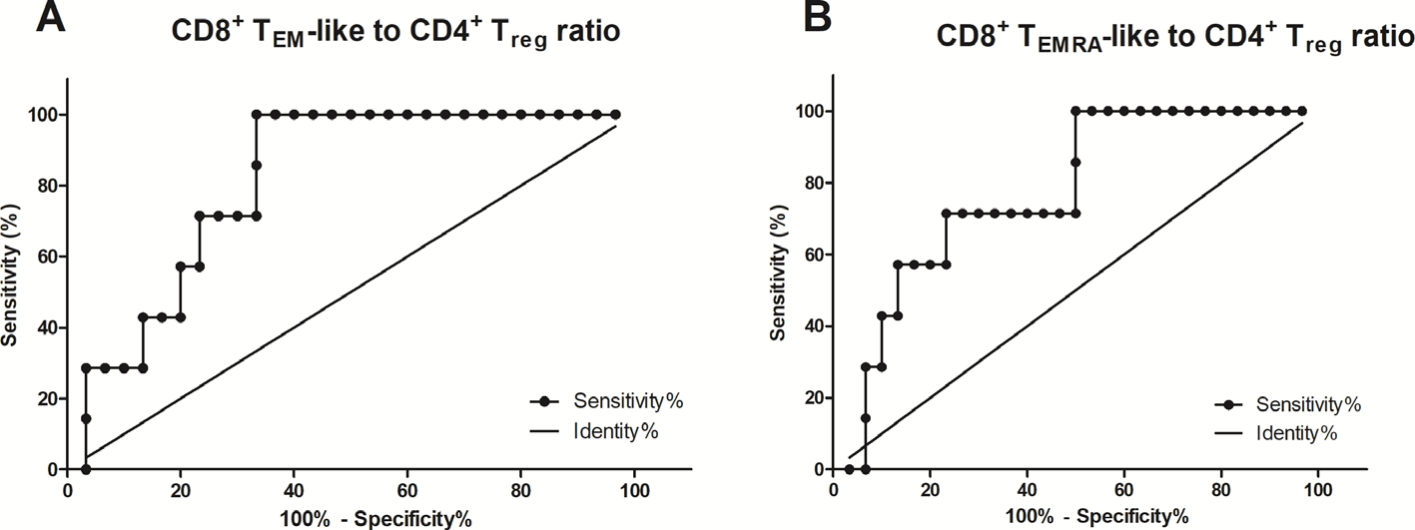
The CD8^+^ T_EM_-like and CD8^+^ T_EMRA_-like cell to CD25^hi^FoxP3^+^CD127^low/–^ CD4^+^ T_reg_ ratios predict impaired fracture healing. **(A)** The ROC curves for the (CD28^–^CD57^–^) CD8^+^ T_EM_-like cell to CD25^hi^FoxP3^+^CD127^low/–^ CD4^+^ T_reg_ ratio in preoperative blood samples (AUC = 0.81, standard error = 0.07, 95% CI 0.67–0.95, n = 37, *P* = 0.01). **(B)** The ROC curves for the (CD28^–^CD57^+^) CD8^+^ T_EMRA_-like cell to CD25^hi^FoxP3^+^CD127^low/–^ CD4^+^ T_reg_ ratio in perioperative blood samples (AUC = 0.7714, standard error = 0.08742, 95% CI 0.60–0.94, n = 38, *P* = 0.027).

We also assessed marker stability using a paired *t*-test and correlation analysis of pre- and postoperative blood samples from 22 patients (**Figure 7**). There were no significant differences between pre- and postoperative levels of CD3^+^ T cells (*P* = 0.37), non-activated (CD28^–^CD57^–^) CD8^+^ T cells (*P* = 0.10), (CD28^–^CD57^+^) CD8^+^ T_EMRA_-like cells (*P* = 0.33), (CCR7^–^CD45RA^–^) CD8^+^ T_naïve_ cells (*P* = 0.79), or (CCR7^–^CD45RA^+^) CD8^+^ T_EMRA_ cells (*P* = 0.12), with correlation analysis confirming a linear relationship between the pre- and postoperative levels. Next, we investigated the potential of the preoperative CD8^+^ T_EMRA_ cells to CD4^+^ T_reg_ ratio as a prognostic marker for delayed fracture healing. We generated ROC curves at each study time point to establish cutoff values (**Figure 8**). We assumed that a potential prognostic marker cannot predict all patients with impaired healing, so we emphasized high specificity over high sensitivity in this analysis. With this prioritization, we aimed to minimize false-positive results and thus to focus on the most relevant cases. Examination of the preoperative (CD28^–^CD57^–^) CD8^+^ T_EM_-like cell to CD4^+^ T_reg_ ratio revealed an acceptable cutoff of > 3.4 for impaired healing, with a sensitivity of 42.9% (95% CI 9.9%–81.6%), a specificity of 86.7% (95% CI 69.3%–96.2%), and a likelihood ratio (LR) of 3.2 (**Figure 8A**). For the preoperative (CD28^–^CD57^+^) CD8^+^ T_EMRA_-like cell to CD4^+^ T_reg_ ratio, we identified a cutoff of > 11.5 for impaired healing. This yielded a sensitivity of 42.8% (95% CI 10%–81.6%), a specificity of 90% (95% CI 73.5%–97.9%), and an LR of 4.3 (**Figure 8B**). For the preoperative (CCR7^–^CD45RA^–^) CD8^+^ T_EM_ cell to CD4^+^ T_reg_ ratio, the cutoff for impaired healing was > 9.1, with a sensitivity of 71.43% (95% CI 29.0%–96.3%), a specificity of 86.7% (95% CI: 69.3%–96.2%), and an LR of 5.36 (**Supplementary Figure S8A**). Finally, for the preoperative (CCR7^–^CD45RA^+^) CD8^+^T_EMRA_ cell to CD4^+^ T_reg_ ratio, we found a cutoff of 9.4 for impaired healing, with a sensitivity of 57.14% (95% CI 18.4%–90.1%), a specificity of 80.0% (95% CI 61.4%–92.3%), and an LR of 2.86 (**Supplementary Figure S8B**). The results suggest that these biomarkers can prospectively identify around 40% of impaired healing cases before surgery, with an acceptable error rate of 10%–15%.

## Discussion

Fracture healing complications, including non-union and delayed healing, pose significant challenges to maxillofacial or musculoskeletal surgical treatment, especially when identified late. Early identification of patients at risk for impaired healing, ideally in a preoperative context, could significantly improve treatment outcomes. However, there are no standardized diagnostic tools that can be used to identify such risks, a situation that complicates the early detection of healing delays (5, 34). Our research has shed light on the dynamics of mandibular fracture healing and supports previous findings from long-bone studies, highlighting the central role of CD8^+^ T_EMRA_ cells and CD4^+^ T_regs_ in the healing process (35). This is rather surprising because, despite some similarities in fracture repair processes between long bones and the mandible, there are substantial differences. The mandible’s neural crest origin, distinct biomechanics, diverse gene expression profiles, and likely unique osteoimmunological microenvironment differ considerably from long bones, particularly in cases of pseudarthrosis following mandibular reconstruction (29, 36).

The immune system–bone interaction is a critical factor in bone homeostasis and repair, particularly given the evolving nature of the immune system over time (3, 37). CD8^+^ (CD28^-^CD57^+^) T_EMRA_-like cells, known for producing pro-inflammatory cytokines such as interferon gamma (IFN-γ) and TNF-α, have been implicated in impairing fracture healing by influencing osteoclastogenesis, extracellular matrix production, angiogenesis, and fibroblastic cell recruitment (3, 28, 38, 39). Although their role has been studied extensively in long bones, our study indicates their potential involvement in mandibular healing, despite differences between the mandible and long bones in their developmental origins and biomechanical properties (40, 41).

We observed higher levels of CD8^+^ T_EMRA_ cells in patients with impaired healing, suggesting their potential as predictive markers for fracture repair complications. Importantly, there was a significant difference in the CD8^+^ T_EMRA_ cell to CD4^+^ T_reg_ ratio between patients with normal and impaired healing, a finding that is consistent with prior studies and that underscores the importance of a balanced immune response for successful bone regeneration (42). Research has highlighted the importance of immune cell dynamics during the different phases of bone healing, especially the intricate relationship between bone cells and B and T cells (25, 43). Reinke et al. (35) demonstrated increased levels of CD8^+^ T_EMRA_ cells in patients with impaired fracture healing, highlighting their negative impact mediated by pro-inflammatory cytokines. Characterization of T_EMRA_ cells based on surface markers such as CD45RA and CCR7 or CD28 and CD57 delineates distinct subsets with different pro-inflammatory potential, further highlighting their role in modulating bone healing (32, 44, 45).

Immune aging is a potential risk factor for impaired healing, as an aged immune system tends to exhibit a more pro-inflammatory phenotype (28). Bucher et al. (38) investigated the effects of immune aging through adaptive immune cell transfer in young animals. They found an aged immune system with a reduced bone-healing capacity. CD4^+^ T_regs_ play a critical role in modulating immune responses and bone homeostasis by inhibiting osteoclast differentiation both *in vitro* and *in vivo* (46–48). Depletion or reduction of CD4^+^ T_regs_ in mouse models has been shown to impair fracture healing, indicating their importance in this process (49). We observed a trend toward increased CD4^+^ T_reg_ levels in patients with normal healing, although it was not statistically significant. Notably, the significant difference we observed in the CD8^+^ T_EMRA_ cell to CD4+ T_reg_ ratio between patients with impaired and normal bone healing aligns with the findings of Schlundt et al. (33), reinforcing the critical role of a balanced effector T cell/T_reg_ response in successful fracture repair. These findings offer promising avenues for therapeutic interventions aimed at modulating T cell responses to improve the fracture healing outcome (50). Nevertheless, challenges remain, including the association between immune senescence and CD57 expression and the need for more comprehensive studies to elucidate the precise mechanisms underlying T cell–mediated modulation of fracture healing (51, 52).

Our study has several methodological limitations, most notably the small sample size, demographic variability, and the inherent challenges in T cell profiling within fracture hematomas. Additionally, the inclusion of patients with varying fracture patterns and dentition, such as angular, corpus, median, and paramedian fractures, introduces a significant confounding factor. Given that these are tooth-bearing areas, disparities in dental and oral health likely influenced the healing outcome. Ideally, we would have focused on a single fracture type with uniform dentition, but this was not feasible. Future research should aim to address these variables to improve the reliability of the results.

To improve comparability across studies, the implementation of standardized T cell profiling protocols is crucial. Therefore, in the present study, we employed protocols that have been used previously and validated in several multicenter studies to ensure consistency and reliability in our immune cell analyses (53). However, advanced imaging techniques, including magnetic resonance imaging (MRI) and micro-CT, offer the potential to characterize immune cell infiltration and bone microarchitecture more precisely during fracture healing (54, 55).

The adoption of standardized scoring systems and the implementation of training programs for radiological assessments are also essential to improve study reproducibility. Furthermore, controlling for confounding factors such as nicotine and alcohol use, as well as patient non-adherence to treatment plans, is critical. While these lifestyle factors were present in our cohort, we did not systematically control them, representing a limitation in our current study design. Understanding the interaction between these factors and immune profiles will be key for developing targeted therapeutic strategies.

Given the exploratory nature of our study, its limitations must be addressed in future longitudinal research to validate the predictive value of the identified immune markers and to investigate potential therapeutic interventions targeting T cell responses during bone healing. Collaborative efforts between orthopedic surgeons, immunologists, and bioengineers will be essential to deepen our understanding of the immune system–bone interface and to develop novel therapeutic approaches (56).

The therapeutic potential of immunomodulatory agents, including anti-inflammatory drugs and T cell–targeted therapies, is an emerging area that could improve fracture healing (38). Further exploration within the fields of osteoimmunology and regenerative medicine could improve fracture repair and also contribute to the management of conditions such as osteoporosis and osteoarthritis (37). Future research should also consider genetic predispositions, comorbidities, and lifestyle factors, as these can significantly impact immune responses and the healing process. Evaluating these factors should contribute to a more comprehensive understanding of the multifactorial nature of fracture healing.

In conclusion, our study underscores the pivotal role of adaptive immunity in mandibular fracture healing. We have elucidated the intricate interplay between immune responses and bone regeneration. Our findings offer valuable insights into how to identify patients at risk of impaired healing prior to surgery, thereby informing surgical strategies and postoperative care, and improving patient outcomes. This research advances maxillofacial surgical and has broader implications for regenerative medicine and orthopedic treatments.

## Data Availability

The raw data supporting the conclusions of this article will be made available by the authors, without undue reservation.
