# Supplementary material for: Prognostic implications of a CD8+ TEMRA to CD4+Treg imbalance in mandibular fracture healing: a prospective analysis of immune profiles
Source: Front Immunol. 2024 Oct 23;15:1476009. doi: 10.3389/fimmu.2024.1476009 (PMC11537918; doi:10.3389/fimmu.2024.1476009)
Supplement: Supplementary file 1 [file DataSheet1.pdf]

Supplementary Material

Prognostic Implications of $CD8^+$ T_{EMRA} to $CD4^+T_{reg}$ Imbalance in Mandibular Fracture Healing: A Prospective Analysis of Immune Profiles

Jan Oliver Voss[1,2#], Fabio Pivetta[3], Aboelyazid Elkilany[3], Katharina Schmidt-Bleek[4,5], Georg N. Duda[4,5], Kento Odaka[6], Ioanna Maria Dimitriou[4,5,7], Melanie Jasmin Ort[4,5,7], Mathias Streitz[8], Max Heiland[1], Steffen Koerdt[1], Simon Reinke[4,5†], Sven Geissler[4,5†]

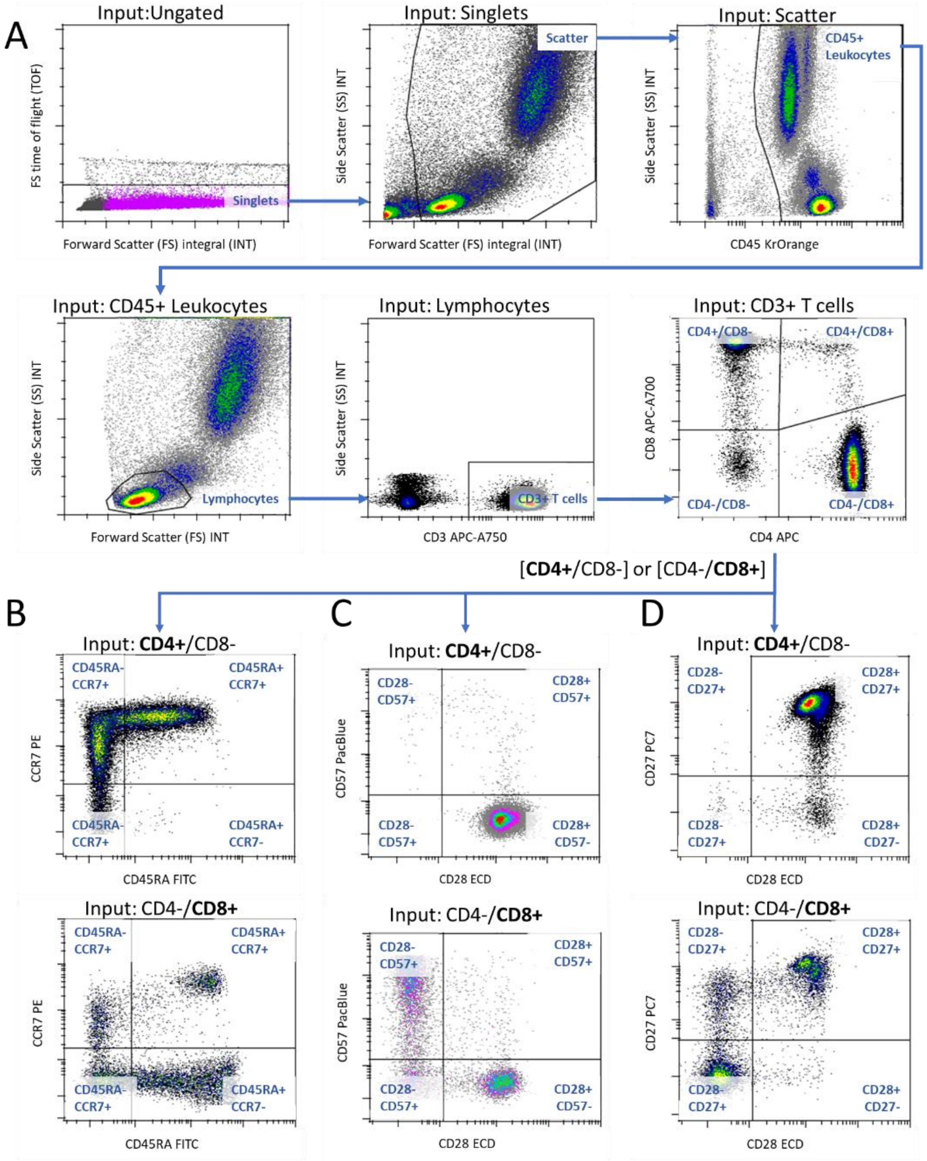

Supplementary Figure 1. Gating strategy for memory T cell subsets & T cell activation. A) The stained lysed (EDTA spiked) whole blood was analyzed with the following gating strategy: non-single events were excluded using forward scatter (FS) time of flight (TOF) versus FS integral (INT), followed by gating of CD45$^+$ leukocytes (anti-CD45 versus sideward scatter [SS] INT). Lymphocytes were gated (FS INT versus SS INT), and CD3+ T cells were identified (anti-CD3 versus SS INT). CD3$^+$ T cells were further divided into CD4$^+$ and CD8$^+$ subsets (anti-CD4 versus anti-CD8). B) CD4$^+$/CD8$^-$ and CD4$^-$/CD8$^+$ subsets were analyzed for naïve (CCR7$^+$/CD45RA$^+$), central memory (CCR7$^+$/CD45RA$^-$), effector memory (T_{EM}, CCR7$^-$/CD45RA$^-$), and terminally differentiated effector memory (T_{EMRA}, CCR7$^-$/CD45RA$^+$) T cells. C) Alternative approach for memory and terminally differentiated subsets using CD28 and CD57 markers: non-activated (CD28$^+$/CD57$^-$), activated (CD28$^+$/CD57$^+$), activated or T_{EM}-like (CD28$^-$/CD57$^-$), and T_{EMRA} (CD28$^-$/CD57$^+$) T cells. D) T cell activation was also assessed by loss of CD27 and/or CD28 expression, identifying CD27$^{-/+}$ and CD28$^{-/+}$ subsets (anti-CD27 versus anti-CD28) within the CD4$^+$/CD8$^-$ and CD4$^-$/CD8$^+$ populations.

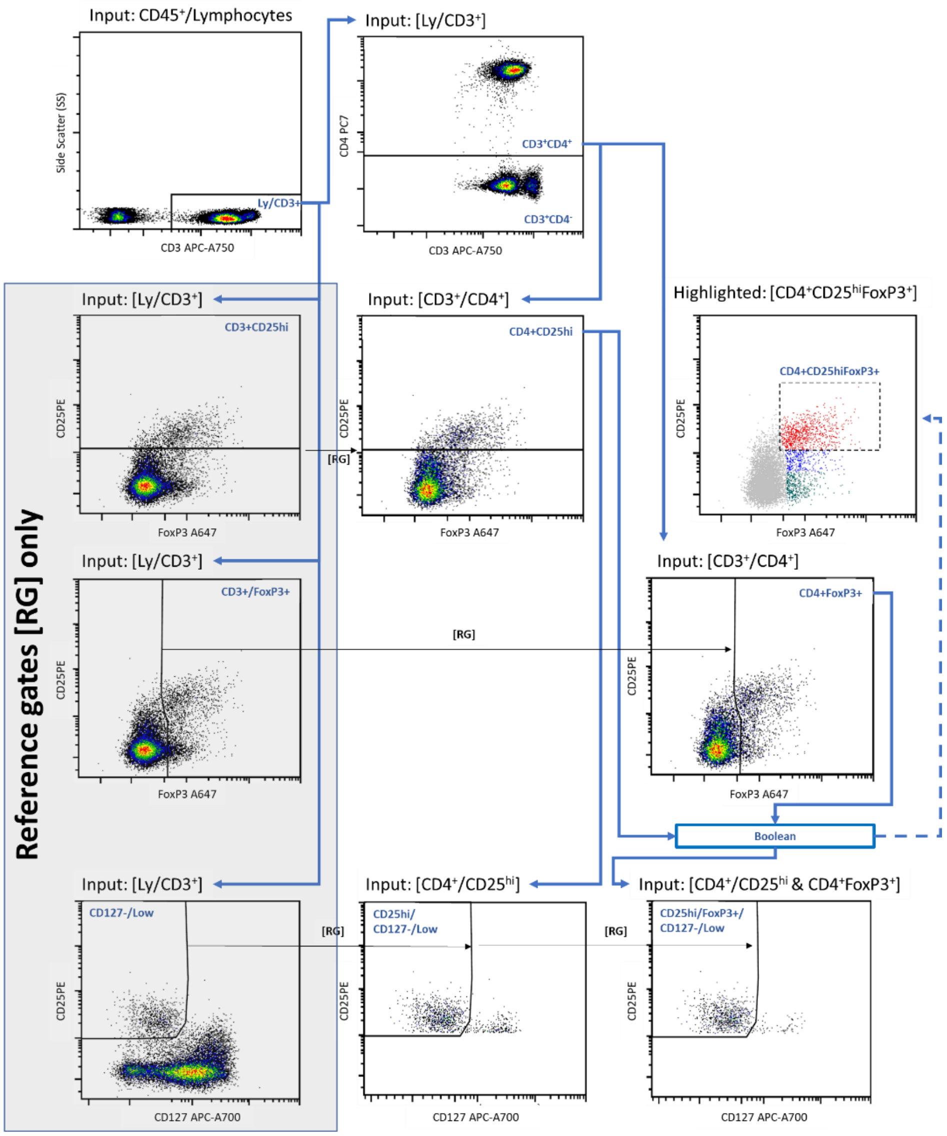

Supplementary Figure 2. Gating strategy for CD4$^+$ regulatory T cell subsets. Stained, lysed whole blood (spiked with EDTA) was analyzed following the gating strategy outlined in Supplementary Figure 1. First, non-single events were excluded using forward scatter (FS) time of flight (TOF) versus FS integral (INT). CD45$^+$ leukocytes were then gated, followed by the selection of lymphocytes. CD3$^+$ T cells were identified using anti-CD3 versus side scatter (SS INT), which were then used to set the reference gates (RG, gray box). These reference gates, created based on the CD3$^+$ T cell population, are indicated by a black arrow and labeled as [RG] in the figure. The CD3$^+$ population was further subdivided into CD4$^+$ and CD4$^-$ subgroups using anti-CD4 versus anti-CD3. Within the CD4$^+$ T cells, subpopulations were distinguished as CD25^{hi} and CD25$^{-/low}$ (anti-CD25 versus anti-FoxP3), with the RG applied. A second plot identified FoxP3$^+$ and FoxP3$^-$ cells (anti-CD25 versus anti-FoxP3). The CD4$^+$CD25^{hi} T cells were further classified into CD127$^{-/low}$ and CD127^{hi} subgroups to identify CD4$^+$CD127$^{-/low}$ regulatory T cells (Tregs). Finally, CD4$^+$CD127$^{-/low}$FoxP3$^+$ Tregs were identified based on the CD4$^+$CD25^{hi}FoxP3$^+$ cell population. This population was determined using a Boolean gate and further subdivided into CD127$^{-/low}$ and CD127^{hi} subpopulations.

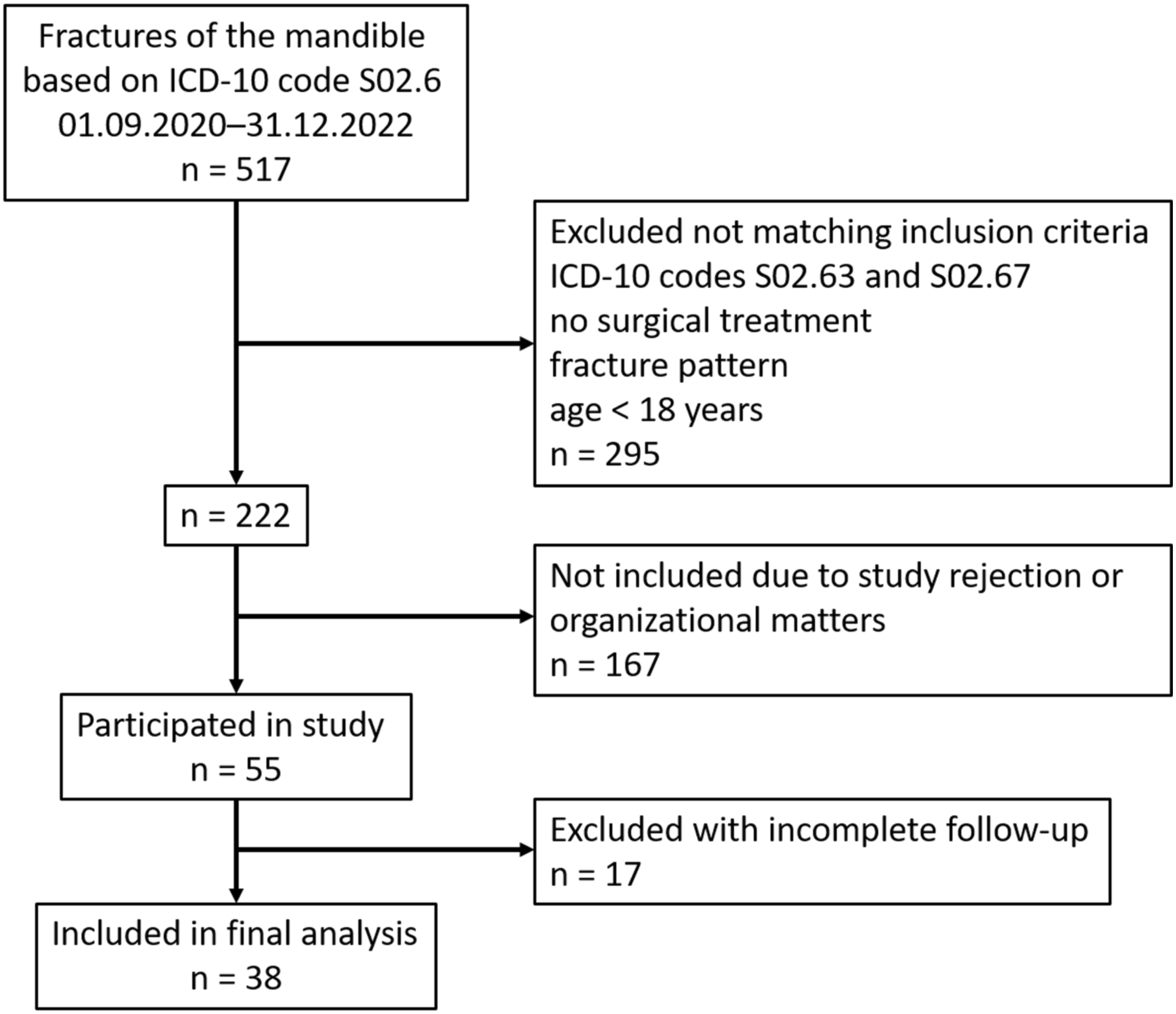

Supplementary Figure 3. Patient inclusion flow chart. From January 1, 2020 to December 31, 2022, a total of 517 cases of mandible fractures, identified by ICD-10 code S02.6, were screened. Following application of the inclusion criteria, 295 cases were excluded. Additionally, 167 cases were excluded due to rejection by the study subjects or organizational issues. Thirty-eight patients qualified for the complete follow-up analysis.

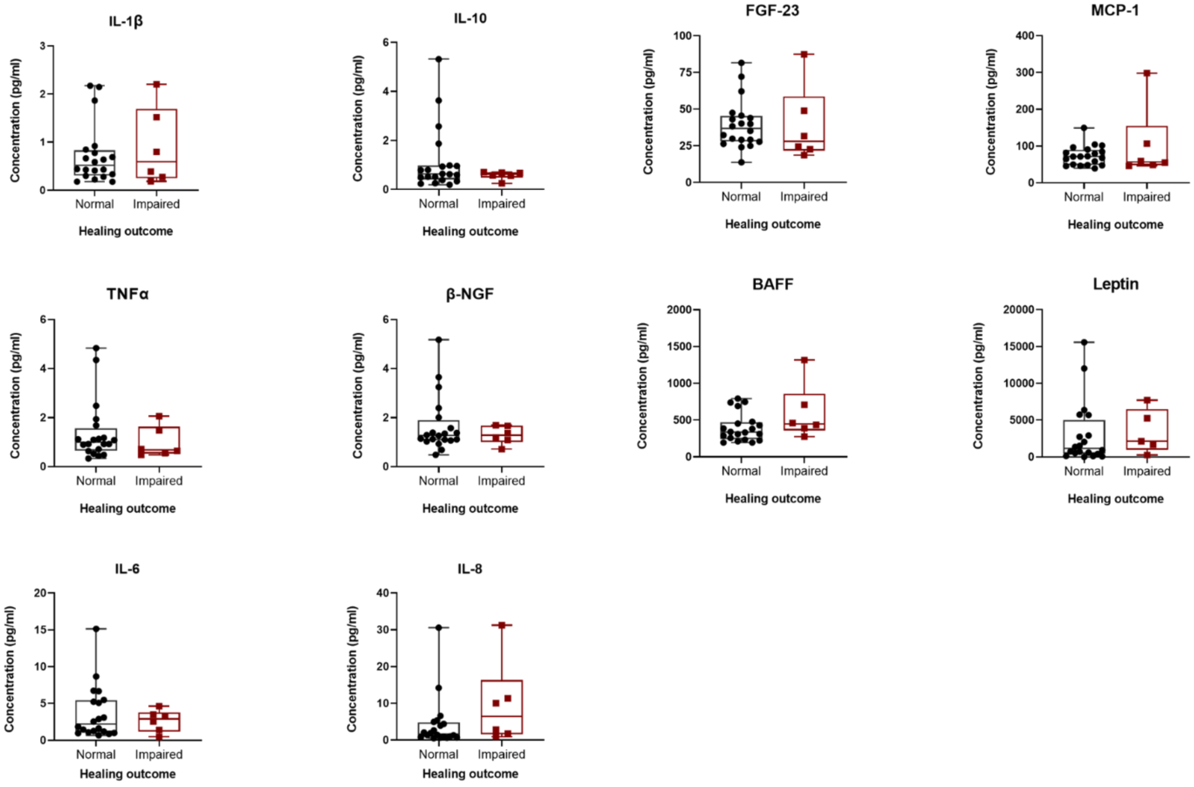

Supplementary Figure 4. Preoperative serum levels of inflammatory and regulatory cytokines in patients with normal versus impaired fracture healing. Serum concentrations of IL-1β, IL-10, FGF-23, MCP-1, TNF-α, β-NGF, BAFF, Leptin, IL-6, and IL-8 were measured using a multiplex immunoassay. No significant differences in cytokine levels were detected between patients with normal healing and those with impaired fracture healing. Data are represented as box plots with individual values.

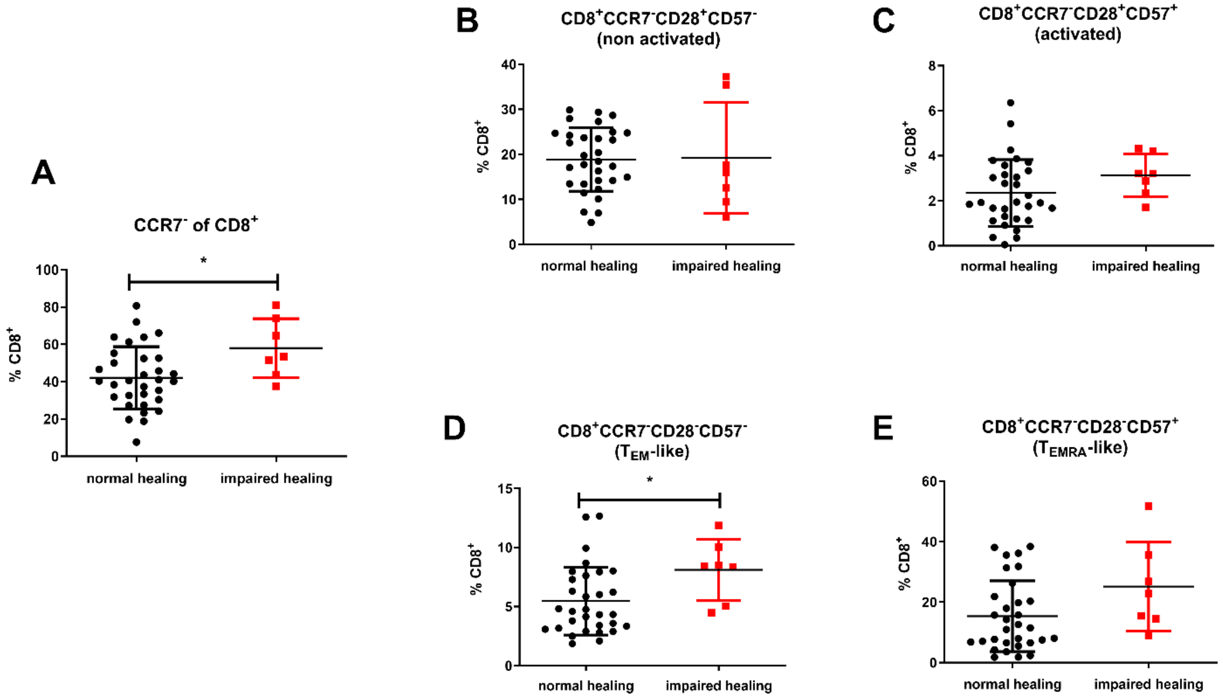

Supplementary Figure 5. Elevated systemic levels of CCR7$^-$CD8$^+$ T cells, (CCR7$^-$CD28$^-$CD57$^-$) CD8$^+$ T_{EM}-like and (CCR7$^-$CD28$^-$CD57$^+$) CD8$^+$ T_{EMRA}-like cells in patients with compromised bone healing. A) Shows CCR7$^-$CD8$^+$ effector compartment. Illustrates B) not activated (CCR7$^-$CD28$^+$CD57$^-$) CD8$^+$ T cells, C) activated (CCR7$^-$CD28$^+$CD57$^+$) CD8$^+$ T cells, D) effector memory-like (T_{EM}-like) (CCR7$^-$CD28$^-$CD57$^-$) CD8+ T cells, and E) terminal differentiated effector memory-like (T_{EMRA}-like) (CCR7$^-$CD28$^-$CD57$^+$) CD8+ T cells. Mann-Whitney U test was employed. *$p < 0.05$.

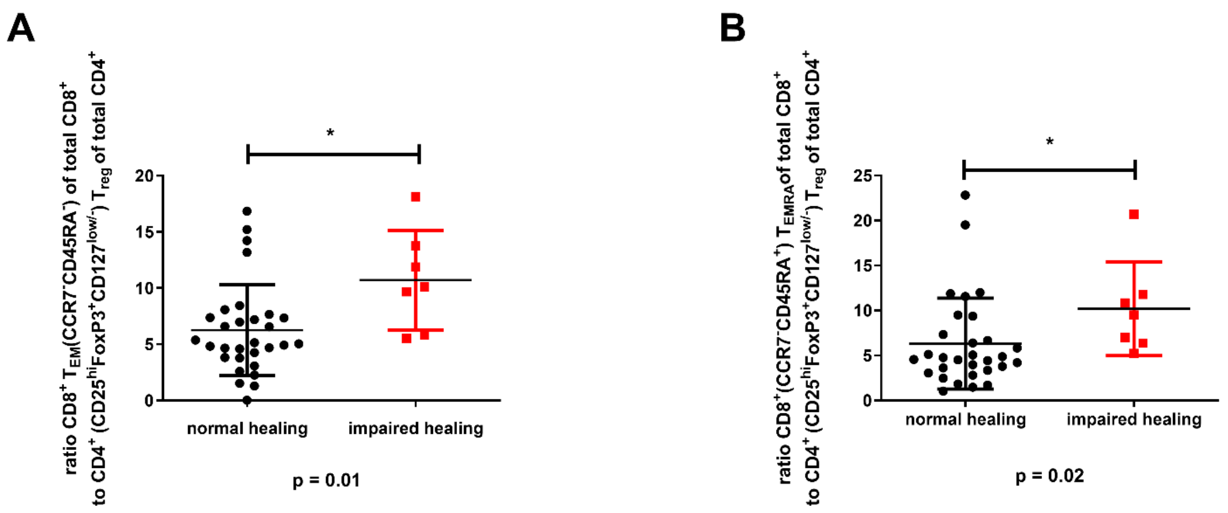

Supplementary Figure 6. Significant association between ($CCR7^-CD45RA^-$) $CD8^+$ T_{EM} or ($CCR7^-CD45RA^+$) $CD8^+$ T_{EMRA} cells to ($CD25^{hi}FoxP3^+CD127^{low/-}$) $CD4^+$ T_{reg} ratios and impaired bone healing. A) Ratio of $CD8^+$ T_{EM} cells to $CD4^+$ Treg. B) Ratio of $CD8^+$ T_{EMRA} of total $CD8^+$ T cells to $CD4^+$ T_{reg}. All data are mean values ± standard deviation in percent of 38 patients. All diagrams include mean values ± standard deviation of the indicated group (n = 38). For A and B, an unpaired two-sided t-test was used. *$p < 0.05$.

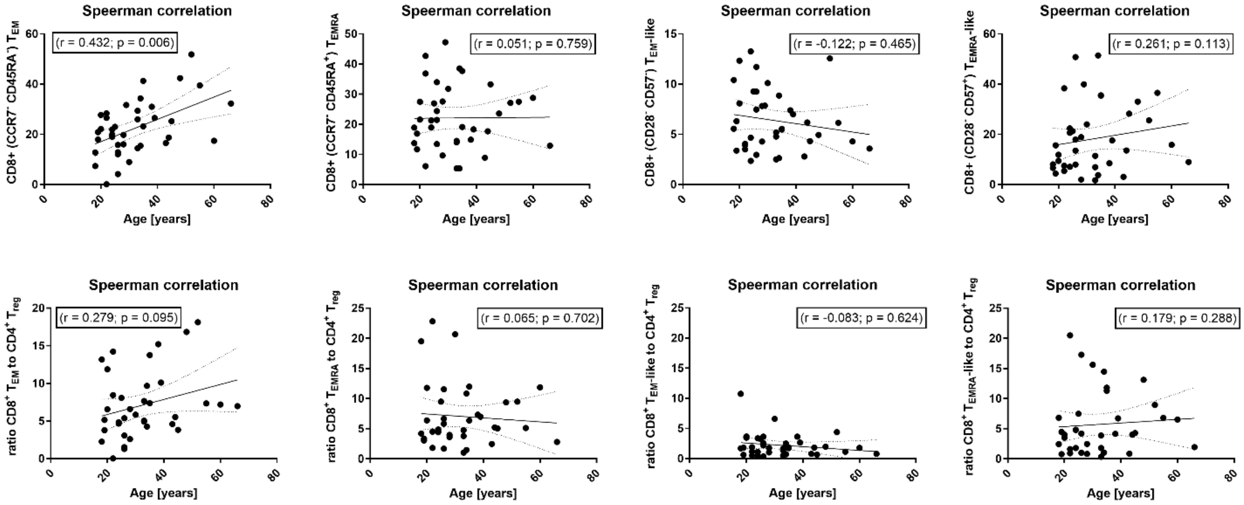

Supplementary Figure 7. Correlation between age and CD8+ T cell subsets. Upper panel) A significant positive correlation was observed between age and $CD8^+$ ($CCR7^-CD45RA^-$) T_{EM} cells. No significant associations were found between age and $CD8^+$ ($CD28^-CD57^-$) T_{EM}-like cells, $CD8^+$ ($CCR7^-CD45RA^+$) T_{EMRA} cells, or $CD8^+$ ($CD28^-CD57^{+}$) T_{EMRA}-like cells. Lower panel) Age did not correlate with the ratios of $CD8^+$ T_{EM} or T_{EMRA} to $CD4^+$ T_{reg} cells.

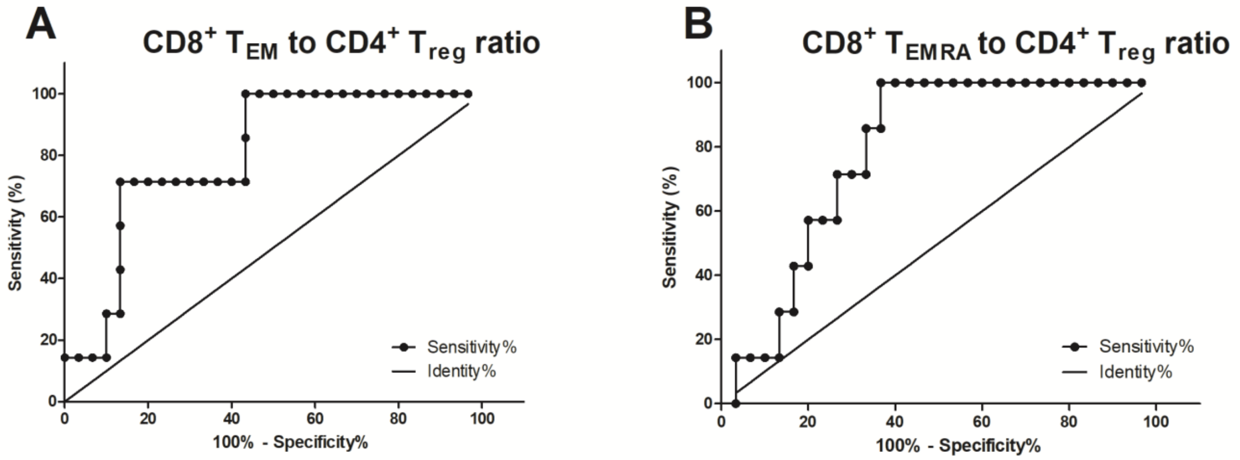

Supplementary Figure 8. $CD8^+$ T_{EM} and $CD8^+$ T_{EMRA} to $CD25^{hi}FoxP3^+CD127^{low/-}$ $CD4^+$ Treg ratios predict impaired fracture healing. A) ROC curves for ($CCR7^-CD45RA^-$) $CD8^+$ T_{EM} to $CD25^{hi}FoxP3^+CD127^{low/-}$ $CD4^+$ T_{reg} ratio in pre-operative blood samples (p = 0.013; AUC = 0.80; Std. Error = 0.08; 95% confidence interval 0.6487 to 0.9608). B) ROC curves for ($CCR7^-CD45RA^+$) $CD8^+$ T_{EMRA} to $CD25^{hi}FoxP3^+CD127^{low/-}$ $CD4^+$ T_{reg} ratio in peri-operative blood samples (p = 0.02; AUC = 0.79; Std. Error = 0.07402, 95% confidence interval 0.6406 to 0.9308).
